# European Delphi consensus on specific training, implementation requirements, and clinical use for the Hugo™ robotic-assisted surgery platform in colorectal procedures

**DOI:** 10.1007/s00384-026-05118-6

**Published:** 2026-03-10

**Authors:** Antonio Arroyo, Orlin Belyaev, Paolo Pietro Bianchi, Pedro Brandão, Pablo Collera, Juan-Manuel Romero-Marcos, Matteo Rottoli, Rebekka Troller, Bert Van Den Bossche, David Daniel Eisinga Zimmerman, Clara Pérez-Esteve, Luis Sánchez-Guillén

**Affiliations:** 1 Department of General Surgery, Colorectal Unit, Elche University Hospital, Elche (Alicante), Spain; 2 Department of Pathology and Surgery, Miguel Hernández University, Ctra. Alicante-Valencia N 332, San Juan de Alicante, Alicante 03550 Spain; 3 Department of General and Visceral Surgery, St. Josef Hospital, Ruhr University Bochum, Gudrunstr, 56, Bochum, 44791 Germany; 4 Department of Surgery, Dipartimento Di Scienze Della Salute, Asst Santi Paolo E Carlo, University of Milan, Milan, Italy; 5 Colorectal Surgery Unit, Unidade Local de Saúde de Santo António, Centro Hospitalar Universitário de Santo António, Porto, 4050-342 Portugal; 6 General Surgery and Digestive System Department, Althaia Foundation, Xarxa Assistencial Universitaria de Manresa, Barcelona, Spain; 7 Department of General Surgery, Hospital Universitari MútuaTerrassa, Terrassa, Barcelona, Spain; 8 Surgery of the Alimentary Tract, IRCCS Azienda Ospedaliero-Universitaria Di Bologna, Bologna, Italy; 9 Department of Surgery, Medway Maritime Hospital, Kent, ME7 5NY UK; 10 General Abdominal Surgery, ASZ Hospital, Merestraat 80, Aalst, 9300 Belgium; 11 Department of Surgery, Elisabeth-TweeSteden Hospital, Tilburg, The Netherlands; 12 Fundación Para El Fomento de La Investigación Sanitaria y Biomédica de La Comunitat Valenciana, Elche (Alicante), Spain; 13 Department of Medical and Surgical Sciences, Alma Mater Studiorum University of Bologna, Bologna, Italy

**Keywords:** Robotic surgery, Colorectal surgery, Hugo™ RAS system, Consensus statement, Delphi study

## Abstract

**Aim:**

A new robotic platform, the Hugo™ robotic-assisted surgery (RAS) system, has been introduced to the market, featuring innovations such as modular arms and an open console, distinguishing it from the Da Vinci system. These differences highlight the need to establish specific, standardized training, credentialing criteria, and clinical guidelines for the use of this platform. To date, this represents the first international expert consensus on the Hugo™ RAS system.

**Methods:**

Eleven European colorectal experts with experience using the Hugo™ RAS platform were invited to participate in this Delphi study. Seventy-seven questions related to this robotic platform were grouped into six domains: (1) required knowledge, (2) technical skills, (3) nontechnical skills, (4) assessment of competency/proficiency during training, (5) credentialing and clinical outcome data, and (6) setups and surgical technique. A three-round Delphi process was conducted. Participants were asked to indicate their agreement or disagreement using a Likert scale (0–5) regarding the proposed themes. Consensus was reached, with a minimum agreement level of 0.80 (80%).

**Results:**

All the experts completed the three Delphi rounds, ensuring a 100% response rate throughout the process. Of the 78 statements evaluated, 33 (42%) achieved consensus agreement (> 80%) and were considered consensus recommendations, while 15 statements showed consensus disagreement (< 20%). The remaining items reflected areas of uncertainty.

**Conclusions:**

The first consensus statement on robotic colorectal surgery with the Hugo™ RAS platform, developed by a European panel of experts, represents an important milestone and provides recommendations for colorectal surgeons considering the adoption of this new robotic platform.

**Supplementary information:**

The online version contains supplementary material available at 10.1007/s00384-026-05118-6.

## Introduction

Robotic-assisted surgery (RAS) continues to transform minimally invasive approaches in colorectal surgery [1, 2]. The Da Vinci (DV) system has historically dominated the robotic landscape, with extensive literature, training pathways, and guidelines, such as the ACPGBI position statement or the ESCP consensus on robotic colorectal training [3, 4]. However, the emergence of new systems, such as Hugo™ RAS (Medtronic), introduces novel challenges and opportunities. Differences in the docking mechanism, arm control, open console, and modular design necessitate tailored recommendations that cannot be directly extrapolated from DV experience. Experience with Hugo™ suggests significant differences in aspects such as ergonomics, positioning, and intraoperative team dynamics [5, 6].

The present study addresses this gap by providing a Delphi consensus among expert European colorectal surgeons. The primary objective was to define expert-driven, consensus-based recommendations for training, mentoring, and credentialing specific to the Hugo™ system. Importantly, the study also sought to enable structured comparisons with existing guidelines and to inform critical needs and best practices for system implementation.

This initiative further focuses on key educational components, such as simulation-based learning, in-person and remote mentoring, and the need to standardize a train-the-trainer model for safe program expansion [7, 8]. The results aim to serve as an evidence-based framework for new adopters, academic programs, and professional bodies worldwide.

## Materials and methods

This study was designed using a modified Delphi methodology to reach expert consensus on training, credentialing, and technical recommendations for the use of the Hugo™ robotic-assisted surgery (RAS) system in colorectal surgery. The Delphi method was selected as an established technique for generating consensus among experts through structured rounds of questioning and controlled feedback.

### Expert panel selection

A panel of 11 European colorectal surgeons from 7 European countries was invited to participate. The selection criteria included recognized clinical expertise in colorectal robotic surgery, active involvement in training programs, and prior experience with at least 50 robotic colorectal procedures using the Hugo™ RAS system, ensuring both geographic and institutional diversity.

### Delphi survey development

The questionnaire was developed by a steering committee with prior experience in robotic colorectal surgery training and Delphi methodology. Statement generation was informed by a targeted review of the literature, existing international robotic training guidelines, and the committee’s clinical and educational expertise. Statements were refined through internal discussion to ensure clarity, relevance, and comprehensive coverage of training, credentialing, and clinical implementation aspects specific to the Hugo™ RAS platform. The preliminary version included 78 statements grouped into six domains:

1. Knowledge requirements
2. Technical skills
3. Nontechnical skills
4. Assessment of training competency
5. Credentialing and outcome evaluation
6. Operating room setup and surgical technique strategies

Statements were evaluated using a 6-point Likert scale (0 = strongly disagree; 5 = strongly agree). Participants could also provide qualitative comments or suggest rewording.

### Delphi process

The consensus process consisted of three iterative rounds. In each round, anonymized responses were aggregated and analyzed. An agreement threshold of ≥ 80% (agree or strongly agree) was selected to define consensus, in line with commonly accepted Delphi methodology and widely used thresholds in surgical education and robotic surgery consensus studies. Statements that reached a predefined threshold of ≥ 80% agreement were accepted as consensus. Those not reaching consensus were revised based on qualitative feedback and resubmitted in subsequent rounds. A response maintained with < 50% on an item in rounds 1 and 2 was deemed impossible to reach consensus, and they were eliminated from the third round. Items with persistent disagreement after round three were excluded or reported descriptively.

Surveys were conducted electronically using secure data collection software. Participants remained blinded to each other throughout, thus minimizing the risk of bias.

### Ethical considerations

Given that the study involved expert opinions without patient-level data, formal ethical approval was not needed. All the participants consented to the anonymous use of their responses for publication purposes.

### Statistical analysis

All survey responses were anonymized and stored securely. The data were exported into Microsoft Excel. Consensus was defined as ≥ 80% of participants rating an item as 4 or 5. Descriptive statistics were used to analyze agreement levels. Trends in agreement across rounds were visualized with histograms.

## Results

### Delphi participation

All 11 invited colorectal surgeons completed the three Delphi rounds, ensuring a 100% response rate throughout the process.

### Overall consensus

Of the 78 initial statements, 33 (42%) achieved consensus agreement (> 80%) and were considered consensus recommendations. Fifteen statements showed consensus disagreement (< 20%) and were therefore considered not recommended by the panel. The remaining statements did not reach consensus and were classified as areas of uncertainty or divergent expert opinion.

#### Domain-specific outcomes

1. Knowledge requirements (Table 1)

**Table 1:** Knowledge required for performing robotic colorectal surgery: recommendations

Item	Description	1 st round % agreement	2nd round % agreement	3rd round % agreement	Status
Q1	A specific robotic training program is required for the use of the Hugo RAS robotic platform	100%	—	—	Accepted
Q2.1	Components of a specific robotic training program with Hugo RAS platform should include e-learning training program prior to laboratory training	72.7%	90.9%	—	Accepted
Q2.2	Components of a specific robotic training program with Hugo RAS platform should include observership: access video-library and real time cases with Touch Surgery platform	63.6%	63.6%	63.6%	Not accepted
Q2.3	Components of a specific robotic training program with Hugo RAS platform should include training program in the handling (placement/docking/use/movements) of robotic arms	100%	—	—	Accepted
Q2.4	Components of a specific robotic training program with Hugo RAS platform should include exercise program with a simulator prior to laboratory training	100%	—	—	Accepted
Q2.5	Components of a specific robotic training program with Hugo RAS platform should include laboratory training with simulation exercises with pig	81.8%	—	—	Accepted
Q2.6	Components of a specific robotic training program with Hugo RAS platform should include laboratory training with cadaver simulation exercises	72.7%	90.9%	—	Accepted
Q2.7	Components of a specific robotic training program with Hugo RAS platform should include evaluation of each training phase (e.g., simulator performance, pig model, cadaveric dissection)	81.8%	—	—	Accepted
Q2.8	Components of a specific robotic training program with Hugo RAS platform should include presence of mentoring/proctor during laboratory training	81.8%	—	—	Accepted
Q2.9	Components of a specific robotic training program with Hugo RAS platform should include telementoring instead of proctor presence in laboratory training	27.3%	9.1%	9.1%	Not accepted
  Strong consensus emerged regarding the foundational knowledge required before Hugo™ training was initiated. Consensus was achieved on 8 of the 10 statements (80%).
  Participants unanimously (100% consensus) agreed in the first round that a specific robotic training program is required to use the Hugo RAS robotic platform. Participants favored a staged curriculum incorporating validated simulator tasks and dry-lab exercises to establish fundamental skills before transitioning to live cases. This program should include e-learning (90% consensus), training programs for handling robotic arms (90% consensus), simulators (90% consensus), and laboratory training in pigs (80% consensus) and cadavers (90% consensus). The presence of mentoring/proctor during laboratory training was recommended (90% consensus) instead of telementoring. Training through observership, through access to video libraries, and real-time cases with a Touch Surgery platform were not considered mandatory. Experts recommended evaluating each training phase (e.g., simulator performance, pig model, cadaveric dissection) (80% consensus).
2. Technical skills (Tables 2 and 3)

**Table 2:** Technical skills required for performing robotic colorectal surgery: recommendations

Item	Description	1 st round % agreement	2nd round % agreement	3rd round % agreement	Status
Q3	Previous laparoscopic experience is recommendable to use robotic surgery with Hugo RAS	90.9%	—	—	Accepted
Q4	Previous robotic experience is recommendable to use robotic surgery with Hugo RAS	0.0%	0.0%	0.0%	Not accepted
Q5	Simultaneous use of the simulator and patient surgery is required during the initial implementation phase of the Hugo RAS robotic program	45.5%	36.4%	36.4%	Not accepted
Q6	In-person intraoperative mentoring is necessary for surgeons performing their initial colorectal robotic cases with Hugo RAS	100%	—	—	Accepted
Q8	After in-person intraoperative mentoring, telementoring intraoperative is recommendable for surgeons performing their next colorectal robotic cases with Hugo RAS	54.5%	45.5%	45.5%	Not accepted
Q10	Performance audits during training in colorectal robotic surgery using the Hugo RAS platform can be conducted through video reviews of recorded surgical procedures	63.6%	72.7%	54.5%	Not accepted
Q12	It is recommended to initiate robotic surgery with Hugo RAS platform by performing simpler procedures to build confidence and proficiency	72.7%	90.9%	—	Accepted
Q13	Before performing colorectal surgery with the Hugo RAS platform, how suitable do you consider it to start with simpler no colorectal procedures, such as robotic cholecystectomy? Please rate each one on a scale from 0 (not at all suitable) to 5 (highly suitable)*	45.5%	45.5%	45.5%	Not accepted
Q16.1	Right hemicolectomy is not suitable for first surgeries with the Hugo RAS platform and should be performed after a learning curve with simpler cases	9.1%	18.2%	18.2%	Not accepted
Q16.2	Right hemicolectomy extended is not suitable for first surgeries with the Hugo RAS platform and should be performed after a learning curve with simpler cases	63.6%	63.6%	72.7%	Not accepted
Q16.3	Laparoscopic ventral rectopexy is not suitable for first surgeries with the Hugo RAS platform and should be performed after a learning curve with simpler cases	18.2%	18.2%	18.2%	Not accepted
Q16.4	Sigmoidectomy (without splenic flexure approach) is not suitable for first surgeries with the Hugo RAS platform and should be performed after a learning curve with simpler cases	27.3%	18.2%	18.2%	Not accepted
Q16.5	Left hemicolectomy (with splenic flexure approach) is not suitable for first surgeries with the Hugo RAS platform and should be performed after a learning curve with simpler cases	81.8%	—	—	Accepted
Q16.6	Recto-sigma resection is not suitable for first surgeries with the Hugo RAS platform and should be performed after a learning curve with simpler cases	27.3%	45.5%	45.5%	Not accepted
Q16.7	High rectal resection is not suitable for first surgeries with the Hugo RAS platform and should be performed after a learning curve with simpler cases	54.5%	72.7%	63.6%	Not accepted
Q16.8	Low rectal resection-AAP is not suitable for first surgeries with the Hugo RAS platform and should be performed after a learning curve with simpler cases	90.9%	—	—	Accepted
Q17	The Hugo RAS robotic system enables the performance of any colorectal surgery that can be performed laparoscopically	100%	—	—	Accepted
Q18.1	Communication with the environment is a key advantage of open and modular robotic systems	90.9%	—	—	Accepted
Q18.2	Surgeon ergonomics are key advantages of open and modular robotic systems	100%	—	—	Accepted
Q18.3	Comfort for bed-assistance is a key advantage of open and modular robotic systems	36.4%	27.3%	27.3%	Not accepted
Q18.4	Versatility (adaptation according to patient and type of surgery) of set-ups is a key advantage of open and modular robotic systems	81.8%	—	—	Accepted
Q18.5	Speed and ease of docking is a key advantage of open and modular robotic systems	54.5%	45.5%	45.5%	Not accepted
Q18.6	Speed and ease of re-docking is a key advantage of open and modular robotic systems	72.7%	72.7%	63.6%	Not accepted
Q18.7	Vision is a key advantage of open and modular robotic systems	81.8%	—	—	Accepted
Q18.8	Autonomy is a key advantage of open and modular robotic systems	72.7%	90.9%	—	Accepted
Q18.9	Range of motion is a key advantage of open and modular robotic systems	63.6%	63.6%	63.6%	Not accepted
Q18.10	Multi-quadrant is a key advantage of open and modular robotic systems	63.6%	72.7%	63.6%	Not accepted
Q18.11	Collisions are key advantages of open and modular robotic systems	27.3%	9.1%	9.1%	Not accepted
Q19	It required a specific structured train-the-trailer (TTT) with Hugo RAS	54.5%	45.5%	45.5%	Not accepted

^*^This item was revised and rephrased in the second round

**Table 3:** Categorical items of block B: technical skills required for performing robotic colorectal surgery

Item	Description		1 st round % agreement	2nd round % agreement	3rd round % agreement
Q7	If you agree that in-person intraoperative mentoring is necessary for surgeons performing their initial colorectal robotic cases with Hugo RAS, for how many cases do you consider it necessary?	1 case	18.2%	14.3%	10.0%
		2–4 cases	72.7%	71.4%	80.0%
		5–7 cases	0.0%	14.3%	10.0%
		> 8 cases	9.1%	0.0%	0.0%
Q9	If you agree that that after in-person intraoperative mentoring, telementoring intraoperative is recommendable for surgeons performing their next colorectal robotic cases with Hugo RAS, for how many cases do you consider it necessary?	1 case	16.7%	0.0%	10.0%
		2–4 cases	66.7%	80.0%	90.0%
		5–7 cases	0.0%	20.0%	0.0%
		> 8 cases	16.7%	0.0%	0.0%
Q11	If you agree that performance audits during training in colorectal robotic surgery using the Hugo RAS platform can be conducted through video reviews of recorded surgical procedures, for how many cases do you consider it necessary?	1 case	0.0%	0.0%	0.0%
		2–4 cases	42.9%	37.5%	71.4%
		5–7 cases	28.6%	50.0%	28.6%
		> 8 cases	28.6%	12.5%	0.0%
Q14	If you have recommended starting robotic cholecystectomy, how many procedures do you consider necessary before starting robotic colorectal surgery with Hugo RAS?	1 case	44.4%	0.0%	14.3%
		2–4 cases	55.6%	100%	85.7%
		5–7 cases	0.0%	0.0%	0.0%
		> 8 cases	0.0%	0.0%	0.0%
Q15	You consider that you acquire the learning curve expert level (trained/capable for more complex colorectal surgeries) with the Hugo RAS platform with:	0–10 cases	0.0%	0.0%	0.0%
		11–20 cases	63.6%	63.6%	45.5%
		21–30 cases	27.3%	36.4%	45.5%
		31–40 cases	9.1%	0.0%	0.0%
		41–50 cases	0.0%	0.0%	9.1%
		> 50 cases	0.0%	0.0%	0.0%
  Consensus was achieved only in 11 of 29 statements (38%). There was widespread agreement in the first round that prior proficiency in laparoscopic colorectal surgery was essential to enter Hugo™ RAS training (90% consensus) but not in robotic surgery (100% consensus). The experts considered (100% consensus) that the Hugo RAS robotic system enables the performance of any colorectal surgery that can be performed laparoscopically.
  In relation to the beginning of the first cases with robotic surgery, experts recommend starting with simple procedures (90% consensus) and with in-person intraoperative mentoring in 2–4 cases (80% consensus). There was no consensus that these proctorizations or performance audits during the initial phase could be conducted via telementoring. Colorectal procedures such as left hemicolectomy with the splenic flexure approach (80% consensus) and low rectal resection (90% consensus) were not suitable for first surgeries with the Hugo RAS platform and should be performed after a learning curve with simpler cases. There was no consensus on the number of cases needed for the learning curve to initiate more complex cases.
  The main advantages of an open and modular robotic system were communication with the environment (90% consensus), surgeon ergonomics (100% consensus), versatility—adaptation according to patient and type of surgery—of setups (80% consensus), vision (80% consensus), and autonomy (90% consensus).
3. Nontechnical skills (Table 4)

**Table 4:** Nontechnical skills required for performing robotic colorectal surgery: recommendation

Item	Description	1 st round % agreement	2nd round % agreement	3rd round % agreement	Status
Q20	Nontechnical skills, such as team working, leadership, situation awareness, decision-making, and communication, are essential components of a colorectal training curriculum for the Hugo RAS platform	54.5%	45.5%	45.5%	Not accepted
  No consensus was reached regarding the inclusion of structured nontechnical skills training such as teamwork, leadership, situational awareness, decision-making, and communication within a Hugo™-specific colorectal training curriculum.
4. Assessment of training competency (Table 5)

**Table 5:** Assessment of competency/proficiency during training in robotic colorectal surgery: recommendation

Question	Description	1 st round % agreement	2nd round % agreement	3rd round % agreement	Status
Q21	With the Hugo RAS platform, the assessment of competency is necessary during training on operative performance and patient clinical outcomes in robotic colorectal surgery	72.7%	90.9%	—	Accepted
  There was a consensus (90%) that the assessment of competency is necessary during training in operative performance and patient clinical outcomes in robotic colorectal surgery.
5. Credentialing and outcome evaluation (Table 6)

**Table 6:** Clinical outcome data registry in robotic colorectal surgery: recommendation

Item	Description	1 st round % agreement	2nd round % agreement	3rd round % agreement	Status
Q22	The Hugo RAS platform is suitable the registration of clinical outcome data in a structured colorectal robotic curriculum	90.9%	—	—	Accepted
Q23.1	Surgical time is one of the critical metrics for evaluating clinical outcomes in robotic colorectal surgery training with the Hugo RAS platform	63.6%	45.5%	45.5%	Not accepted
Q23.2	Intraoperative complications are one of the critical metrics for evaluating clinical outcomes in robotic colorectal surgery training with the Hugo RAS platform	100%	—	—	Accepted
Q23.3	Conversion rates are among the critical metrics for evaluating clinical outcomes in robotic colorectal surgery training with the Hugo RAS platform	90.9%	—	—	Accepted
Q23.4	Anastomotic leak rate is one of the critical metrics for evaluating clinical outcomes in robotic colorectal surgery training with the Hugo RAS platform	45.5%	45.5%	45.5%	Not accepted
Q23.5	Postoperative morbidity is one of the critical metrics for evaluating clinical outcomes in robotic colorectal surgery training with the Hugo RAS platform	81.8%	—	—	Accepted
Q23.6	Reintervention is one of the critical metrics for evaluating clinical outcomes in robotic colorectal surgery training with the Hugo RAS platform	72.7%	72.7%	90.9%	Accepted
Q23.7	Lymph node yield is one of the critical metrics for evaluating clinical outcomes in robotic colorectal surgery training with the Hugo RAS platform	72.7%	72.7%	72.7%	Not accepted
Q23.8	R1 resections are among the critical metrics for evaluating clinical outcomes in robotic colorectal surgery training with the Hugo RAS platform	90.9%	—	—	Accepted
  Six of the nine statements (67%) reached consensus. Consensus (90%) was reached on the importance of a formal structured colorectal robotic curriculum with the Hugo RAS platform based on the following critical metrics for evaluating clinical outcomes: intraoperative complications (consensus 100%), conversion rates (consensus 90%), postoperative morbidity (consensus 80%), reintervention (consensus 90%), and R1 resections (consensus 90%).
6. Setup and surgical technique (Tables 7 and 8)

**Table 7:** SET-UP and surgical technique

Item	Description	1 st round % agreement	2nd round % agreement	3rd round % agreement	Status
Q24	I use the setup configurations recommended by the manufacturer	18.2%	9.1%	9.1%	Not accepted
Q25	The modularity of the arms allows me to modify and adapt the setup	100%	—	—	Accepted
Q26.1	When modifying the setup configurations, I base them on the patient’s height	72.7%	72.7%	90.9%	Accepted
Q26.2	When modifying the setup configurations, I base them on the patient’s weight	90.9%	—	—	Accepted
Q26.3	When modifying the setup configurations, I base them on the need for extended surgeries (flexure access)	90.9%	—	—	Accepted
Q26.4	When modifying the setup configurations, I base them on multiquadrant surgery	90.9%	—	—	Accepted
Q28.1	In multiquadrant extended right hemicolectomy (with hepatic flexure descent and access media colic vessels), I perform patient position change (Trendelenburg/anti-Trendelenburg)	36.4%	18.2%	18.2%	Not accepted
Q28.2	In multiquadrant extended right hemicolectomy (with hepatic flexure descent and access media colic vessels), I perform new trocar placement	18.2%	9.1%	9.1%	Not accepted
Q28.3	In multiquadrant extended right hemicolectomy (with hepatic flexure descent and access media colic vessels), I perform re-docking	27.3%	0.0%	0.0%	Not accepted
Q28.4	In most procedures, I do not perform any changes during multiquadrant extended right hemicolectomy (with hepatic flexure descent and access to the middle colic vessels)	72.7%	90.9%	—	Accepted
Q29.1	In multiquadrant left hemicolectomy or rectum surgery requiring splenic flexure descent, I perform patient position change (Trendelenburg/anti-Trendelenburg)	36.4%	18.2%	18.2%	Not accepted
Q29.2	In multiquadrant left hemicolectomy or rectum surgery requiring splenic flexure descent, I perform new trocar placement	18.2%	9.1%	9.1%	Not accepted
Q29.3	In multiquadrant left hemicolectomy or rectum surgery requiring splenic flexure descent, I perform re-docking	63.6%	45.5%	45.5%	Not accepted
Q29.4	In most procedures, I do not perform any changes during multiquadrant left hemicolectomy or rectum surgery requiring splenic flexure descent	54.5%	45.5%	45.5%	Not accepted
Q30.1	In multiquadrant left hemicolectomy surgery or recto-sigma resection without the need for splenic flexure descent, I perform patient position change (Trendelenburg/anti-Trendelenburg)	27.3%	18.2%	18.2%	Not accepted
Q30.2	In multiquadrant left hemicolectomy surgery or recto-sigma resection without the need for splenic flexure descent, I perform new trocar placement	18.2%	9.1%	9.1%	Not accepted
Q30.3	In multiquadrant left hemicolectomy surgery or recto-sigma resection without the need for splenic flexure descent, I perform re-docking	45.5%	9.1%	9.1%	Not accepted
Q30.4	In most procedures, I do not perform any changes during multiquadrant left hemicolectomy surgery or recto-sigma resection without the need for splenic flexure descent	81.8%	—	—	Accepted

**Table 8:** Categorical items of block F: SET-UP and surgical technique

Item	Description		1 st round % agreement	2nd round % agreement	3rd round % agreement
Q27.1	In the right hemicolectomy, the placement of the robotic arms is done on the sides according to the configuration	2 right-2 left	54.5%	54.5%	54.5%
		3 left-1 right	27.3%	27.3%	27.3%
		Other	18.2%	18.2%	18.2%
Q27.2	In the left colon, the placement of the robotic arms is done on the sides according to the configuration	2 right-2 left	63.6%	63.6%	63.6%
		3 left-1 right	27.3%	27.3%	27.3%
		Other	9.1%	9.1%	9.1%
Q27.3	In the recto-sigma, the placement of the robotic arms is done on the sides according to the configuration	2 right-2 left	54.5%	54.5%	54.5%
		3 left-1 right	27.3%	27.3%	27.3%
		2 left-1 right-1 between beds	9.1%	9.1%	9.1%
		Other	9.1%	9.1%	9.1%
Q27.4	In the rectum, the placement of the robotic arms is done on the sides according to the configuration	2 right-2 left	63.6%	54.5%	54.5%
		3 left-1 right	9.1%	9.1%	9.1%
		2 left-1 right-1 between beds	18.2%	27.3%	27.3%
		Other	9.1%	9.1%	9.1%
  A consensus was reached (80%) that the experts did not use the setups recommended by the manufacturer. The modularity of the arms allows modification and adaptation of the setup (consensus 100%). They modified the setup configurations based on the patient’s height, patient’s weight, need for extended surgeries (flexure access), and multiquadrant surgery, all of which had a 90% consensus. Among the different configurations, there was no consensus on the placement of the robotic arms in any type of colectomy.
  Regarding modifications during surgery in the patient position or the placement of additional trocars or redocking, in the multiquadrant procedure of extended right hemicolectomy or in multiquadrant left hemicolectomy surgery or recto-sigma resection without the need for splenic flexure descent, a consensus was reached that no changes were made (90% and 80%, respectively), but in multiquadrant left hemicolectomy or rectum surgery requiring splenic flexure descent, there was no consensus.

## Discussion

In recent years, there has been a growing adoption of robotic surgery worldwide, with a notable increase in the acquisition of new robotic platforms and in the number of procedures performed. However, this expansion has not been without its challenges, especially in regard to standardized training and identifying best practices for these new robotic platforms. For this reason, evaluating the evidence regarding certain essential training elements when a formal training scheme is being established is important [9].

The Delphi method is a robust and widely used methodology to establish guidelines in fields where empirical evidence is still limited or developing and is especially relevant for new surgical technologies such as the Hugo RAS. Previous studies have successfully used this Delphi methodology to define training curricula in robotic surgery [3, 8–12]. These precedents establish a solid foundation for the methodology employed in the present consensus on Hugo™ RAS, validating the Delphi approach as an effective tool for expert-based decision-making in the field of robotic surgery. Current clinical evidence regarding the use of the Hugo™ RAS system in colorectal surgery is largely limited to early series, observational studies, and single-center experiences. While feasibility and acceptable short-term outcomes have been reported, robust comparative data and long-term oncological outcomes remain scarce [2, 5, 6]. This gap highlights the preliminary nature of the present consensus and the need for prospective studies and registry-based analyses.

This European Delphi consensus represents the first comprehensive effort to define platform-specific requirements for the implementation and clinical integration of the Hugo™ robotic-assisted surgery (RAS) system in colorectal procedures. Across three Delphi rounds, consensus recommendations were achieved for 33 statements, while 15 statements showed consensus disagreement and several others reflected areas of uncertainty, underscoring the evolving nature of best practices for this emerging robotic platform. All experts were accredited through a common training and education model at Hugo RAS, which homogenizes the start of the program and shows the evolution of these programs according to experience. The sample of experts combines naïve/robotic experts, hospitals of different sizes, and geographical diversity, which gives added value for the transfer of knowledge from this Delphi.

Unlike previous studies that focused exclusively on the DV system, such as those published by the European Society of Coloproctology (ESCP) [4] and the Association of Coloproctology of Great Britain and Ireland (ACPGBI) [3], our study addresses the need for distinct recommendations tailored to the modular and open-console nature of the Hugo™ platform. Our consensus reflects the ongoing evolution in adapting surgical education and surgical planning to newer, more flexible platforms such as Hugo™, which diverges significantly in design and workflow compared with DV. Hugo-specific analysis highlights key distinctions, such as platform configuration, console ergonomics, and arm docking strategies, which require unique competencies not previously addressed in DV-based curricula [5, 6]. This highlights the nontransferability of certain procedural steps and the necessity of dedicated Hugo™ RAS programs.

With respect to knowledge requirements, the ESCP guidelines [4] recommend that robotic platform training is essential to patient safety and therefore should be used in a structured colorectal robotic training curriculum. Hugo’s consensus sets out recommendations very similar to those of the DV platform [4, 11], with a staged curriculum incorporating validated simulator tasks, dry-lab exercises, and hands-on training (wet-lab or cadaveric) to establish fundamental skills before transitioning to live cases. However, although e-learning could be used to deliver colorectal robotics content [12], in this consensus, the possibility of telementoring in this phase of training was not considered adequate and was not sufficiently mature to recommend it. New evidence is needed to evaluate the validity of this e-learning educational content and surgeons’ or trainees’ acceptability and accessibility [4].

In relation to *technical skills*, several topics were discussed. With the same opinion of both ESCP and ACPGBI, our panel strongly endorsed the need for in‑person intraoperative mentoring during initial colorectal cases (100% agreement). Recently, the possibility of telementoring in this training phase has begun to be explored, with promising results in the mentoring of surgery [13, 14], but there is currently no strong evidence or recommendation to consider it of choice.

A very important aspect of naïve robotic surgeons is the choice of patient or selection of surgical procedures at the beginning of the robotic program. A recent systematic review revealed that robotic colorectal surgery involves a measurable learning curve that affects both technical and patient-centered outcomes [15]. Our panel advised us to start with easier procedures to build confidence and proficiency. The pattern of responses regarding case selection also mirrors guideline recommendations: right hemicolectomy and sigmoidectomy without splenic flexure mobilization were considered ideal procedures, whereas left hemicolectomy with flexure mobilization and low rectal resection/abdominoperineal resection were clearly regarded as inappropriate for the very first cases, which is consistent with the notion that multiquadrant and low pelvic resections should be reserved for later in the learning curve. Similarly, there was agreement that the Hugo RAS system can be used for any procedure that is currently feasible laparoscopically and broad support for the core advantages of an open, modular platform, particularly enhanced communication, surgeon ergonomics, versatility of setups, vision, and perceived autonomy.

Another widely discussed aspect is the beginning of a robotic surgery program with no previous experience in laparoscopic surgery [16, 17]. Although ESCP states that prior laparoscopic experience is not essential, most of the respondents in our study felt that laparoscopic experience is, in fact, recommended before adopting Hugo RAS. Conversely, there was no support for requiring previous robotic experience, which is in keeping with both guidelines emphasizing structured curricula and fellowships rather than prior exposure to another robotic platform. Specific programs and training routes may remain to be defined based on previous experience in laparoscopic and/or other robotic platforms. Perhaps the Hugo robotic platform with open console and modular arms with trocar placement, which is more versatile and closer to laparoscopic surgery, leads to this consensus conclusion.

Finally, the panel’s view that expert-level proficiency can be achieved in the range of 11–30 cases on Hugo RAS is understandable given the conceptual similarities of open and modular console to laparoscopic surgery. However, the numerical findings suggest an optimistic, fast learning curve compared with some of the longer case volumes reported in the literature [18, 19], and they might suggest a potential need to tailor training frameworks to accommodate emerging robotic technologies that differ in interface, setup, and team dynamics from the DV system. This estimate likely reflects the number of procedures required to achieve safe autonomous practice rather than true expert-level proficiency and suggests the need to distinguish early autonomy from advanced expertise when designing training and credentialing frameworks.

A noteworthy difference emerged regarding *nontechnical skills*. The ESCP guidelines make a specific recommendation: training in nontechnical skills could be considered part of the training curriculum for robotic colorectal surgery. It explicitly identifies teamwork, leadership, situational awareness, decision-making, and communication as fundamental elements for patient safety, stressing that many complications in minimally invasive surgery are associated with failures in these competencies rather than with purely technical deficits. Even so, the ESCP guidelines acknowledge that specific empirical evidence on colorectal robotics is scarce and call for more studies comparing programs with and without structured training in nontechnical skills. In the same way, Keating [2] and Gomez [7] published two international Delphi studies that reported that in robotic surgeries with DV, nontechnical skills are key perceived benefits of RAS systems and are considered a fundamental part of robotic programs. However, our Delphi panel failed to reach a consensus on specific nontechnical training requirements for Hugo™. Importantly, the lack of consensus regarding nontechnical skills should not be interpreted as questioning their importance. Rather, it reflects uncertainty among experts on how these skills should be formally structured, assessed, and integrated into a Hugo™-specific training curriculum. The open-console design of the Hugo™ RAS system may facilitate real-time communication and teamwork, potentially influencing expert perceptions during this early phase of adoption [20, 21].

With respect to the recommendation for *credentialing and outcome evaluation*, our consensus revealed the importance of formal structured colorectal robotic curricula with the Hugo RAS platform based on specific critical metrics (intraoperative complications, conversion rates, postoperative morbidity, reintervention, and R1 resections) for evaluating clinical outcomes. The lack of consensus on other metrics such as operative time or anastomotic leak rate may reflect their dependence on case mix, procedural complexity, and institutional benchmarking variability, as well as their limited discriminatory value during early implementation phases. However, this statement, which aligns with most of the publications on the subject [4, 22], is based on expert opinions since there are currently no studies that demonstrate better results with the recording of outcomes.

A key area of divergence between this consensus and prior DVs is surgical setup and docking. DV systems that operate with a fixed cart with trocar positioning and patient setup are largely standardized and require limited adjustments in most colorectal procedures [23]. In contrast, our panel revealed considerable variability in the configurations of Hugo’s™-dependent surgeons. There was no consensus on a single preferred setup, and it was considered procedure-, patient-, and anatomy-dependent. This is reflected in the different publications on Hugo RAS, where different setups can be used for the same procedure, and with the same setup, it can be used in different procedures, even when it is able to work in multiquadrant accesses without changing the setup [2, 5, 24, 25]. This flexibility of this robotic platform, while advantageous in theory, introduces variability in training, prompting our recommendation for platform-specific docking simulations and practical scenarios. From a practical implementation standpoint, new adopters of the Hugo™ RAS platform may benefit from starting with a baseline manufacturer-recommended setup, followed by progressive adaptation based on patient height, body habitus, and multiquadrant requirements. Rather than a single optimal configuration, a decision-rule approach may help centers safely evolve their setup strategies as experience increases.

This study is limited by the relatively small, exclusively European expert panel composed of early adopters with substantial experience using the Hugo™ platform. While this ensures informed expert input, it may limit generalizability to centers initiating robotic programs from scratch or operating in different healthcare systems. Additionally, consensus was achieved for only 42% of statements, reflecting the early stage of platform adoption and the ongoing evolution of best practices. In summary, this European Delphi consensus for the Hugo™ RAS platform in colorectal surgery represents a significant step towards standardizing and optimizing the training and clinical use of this emerging technology. Like other Delphi consensuses in robotic surgery, it seeks to establish guidelines based on expert opinion to address the lack of structured training and ensure the safety and efficacy of procedures. The findings of this study will be crucial in developing training programs that adequately prepare surgeons and surgical teams for the growing demand for robotic procedures in the colorectal field. Continued research and validation following the implementation of these guidelines will be essential to consolidate the role of Hugo™ RAS in modern colorectal surgery and ensure that its adoption is carried out responsibly and with the highest quality standards.

## Supplementary information

Below is the link to the electronic supplementary material.

ESM 1 (DOCX 225 KB)

## Data Availability

The data that support the findings of this study are available from the corresponding author upon reasonable request.
