# Supplementary figures and images for: European Delphi consensus on specific training, implementation requirements, and clinical use for the Hugo™ robotic-assisted surgery platform in colorectal procedures
Source: Int J Colorectal Dis. 2026 Mar 10;41(1):77. doi: 10.1007/s00384-026-05118-6 (PMC12979304; doi:10.1007/s00384-026-05118-6)

**VISUAL ANALYSIS OF LIKERT SCALE DATA**


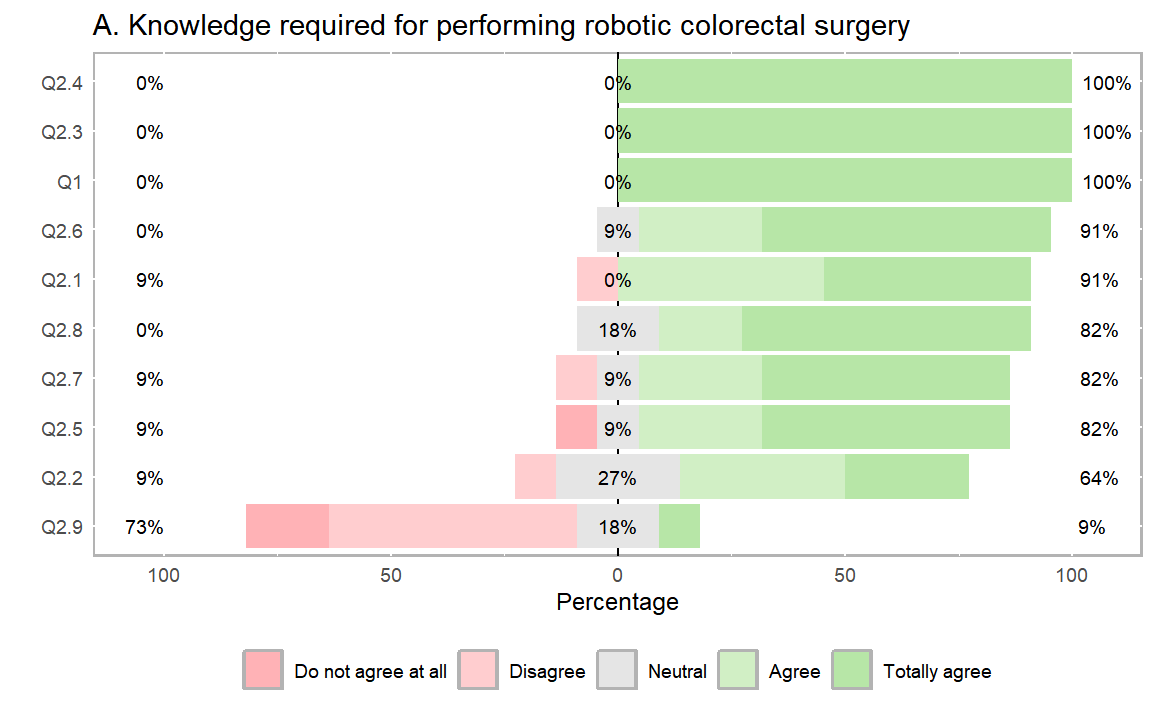


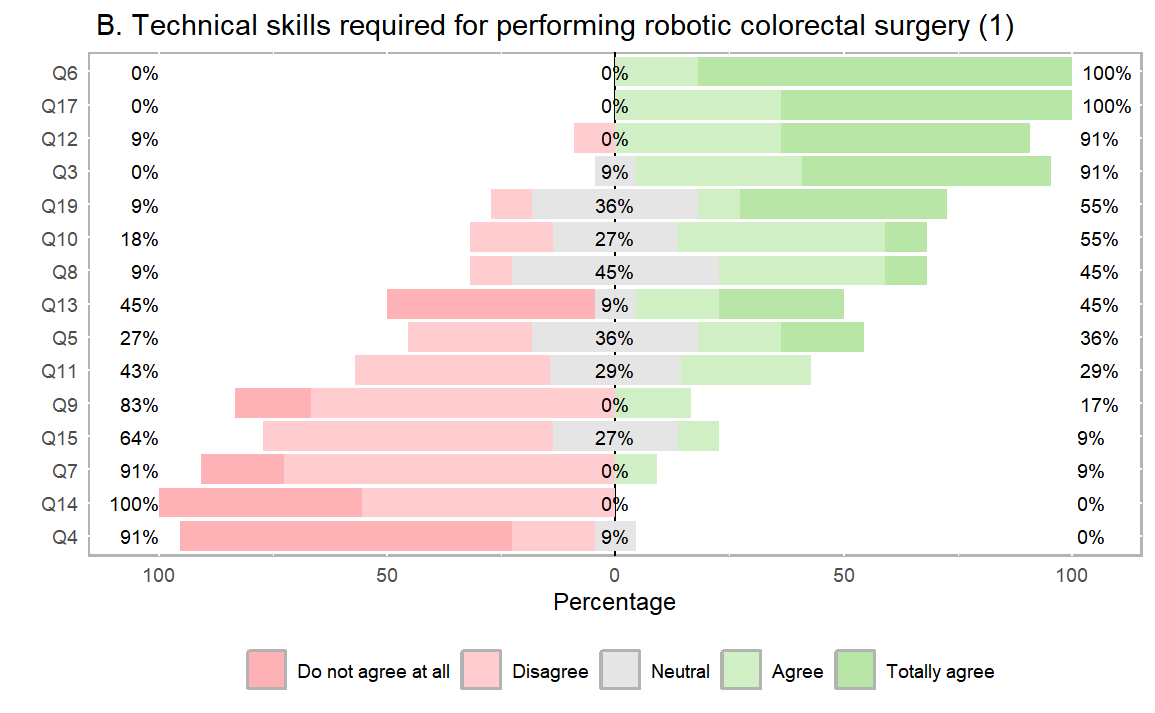


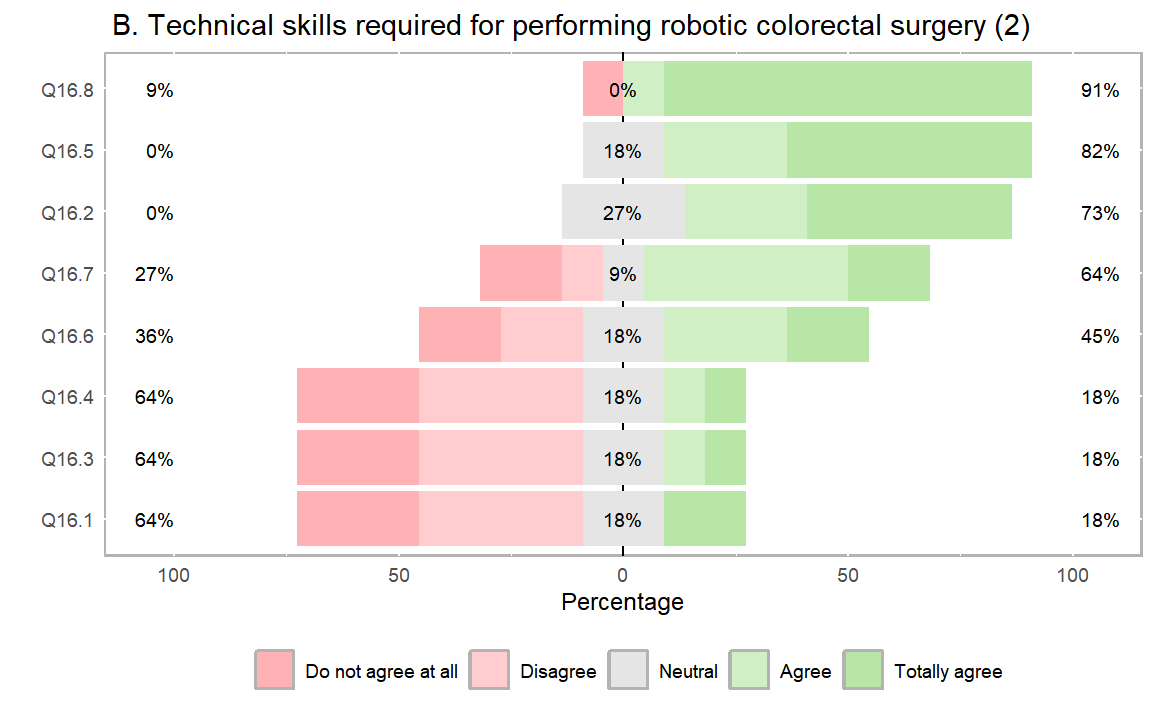


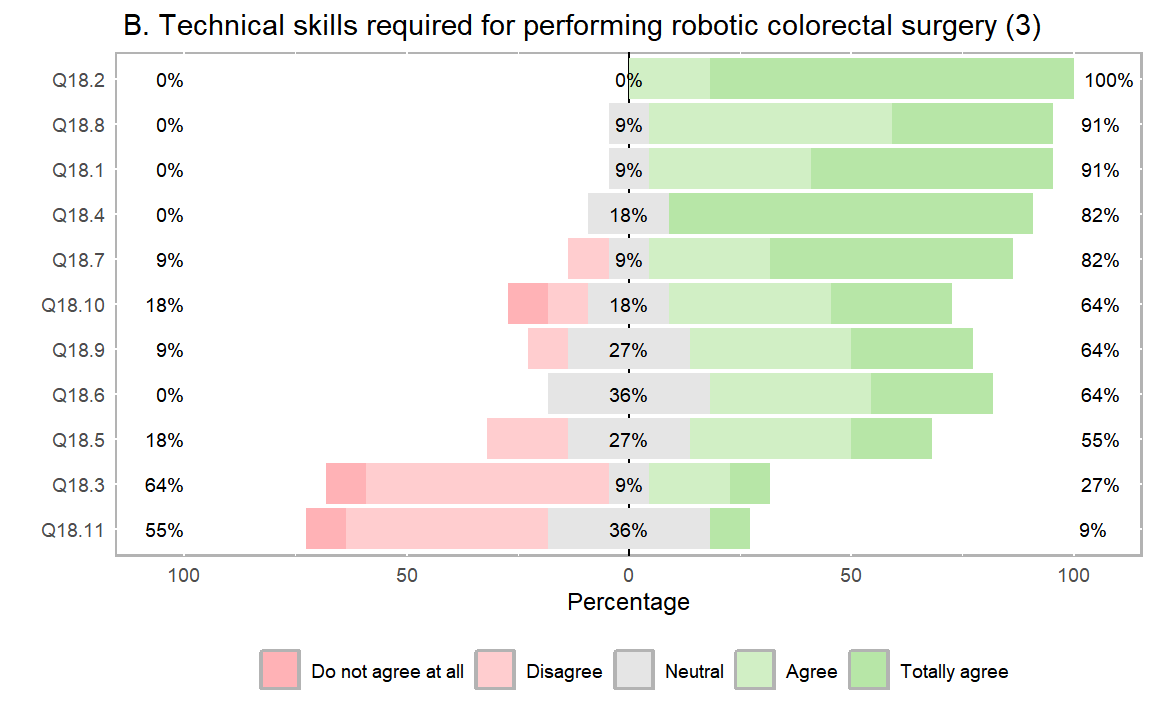


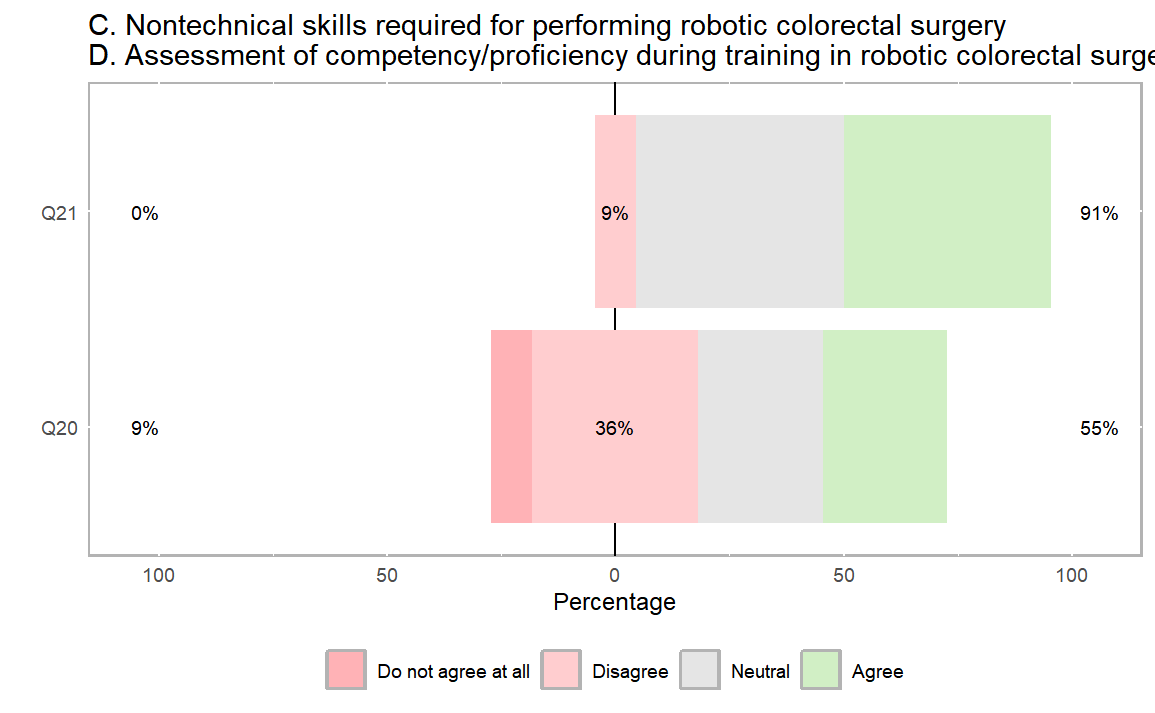


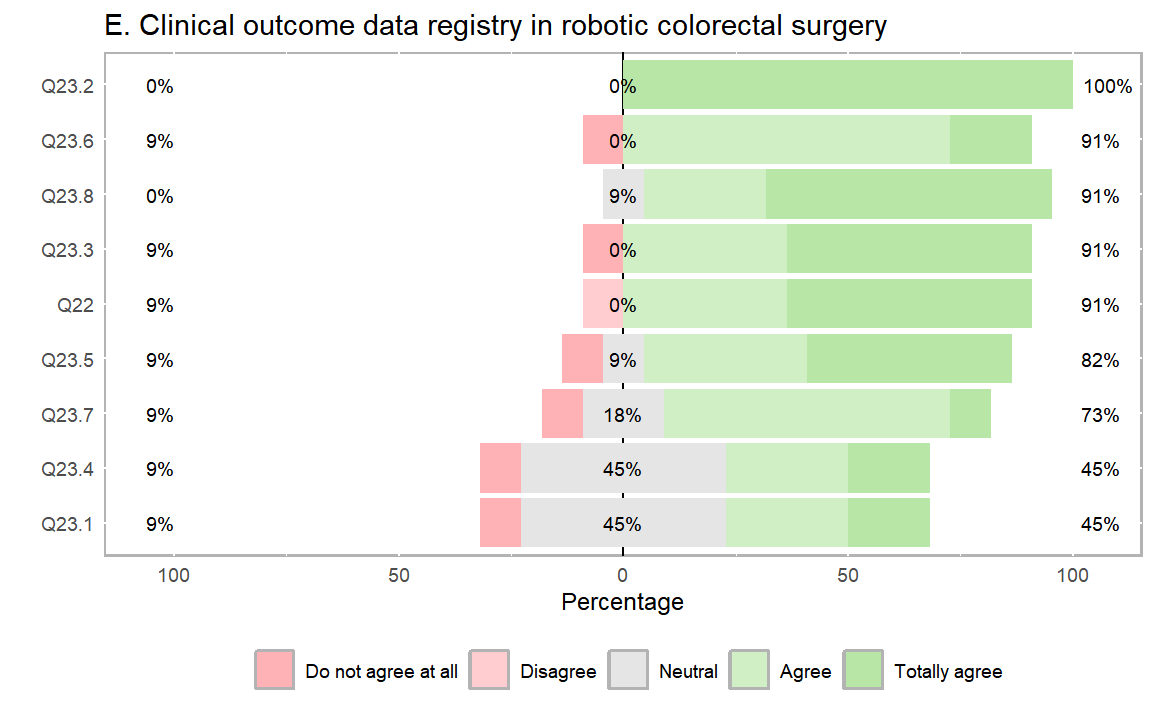


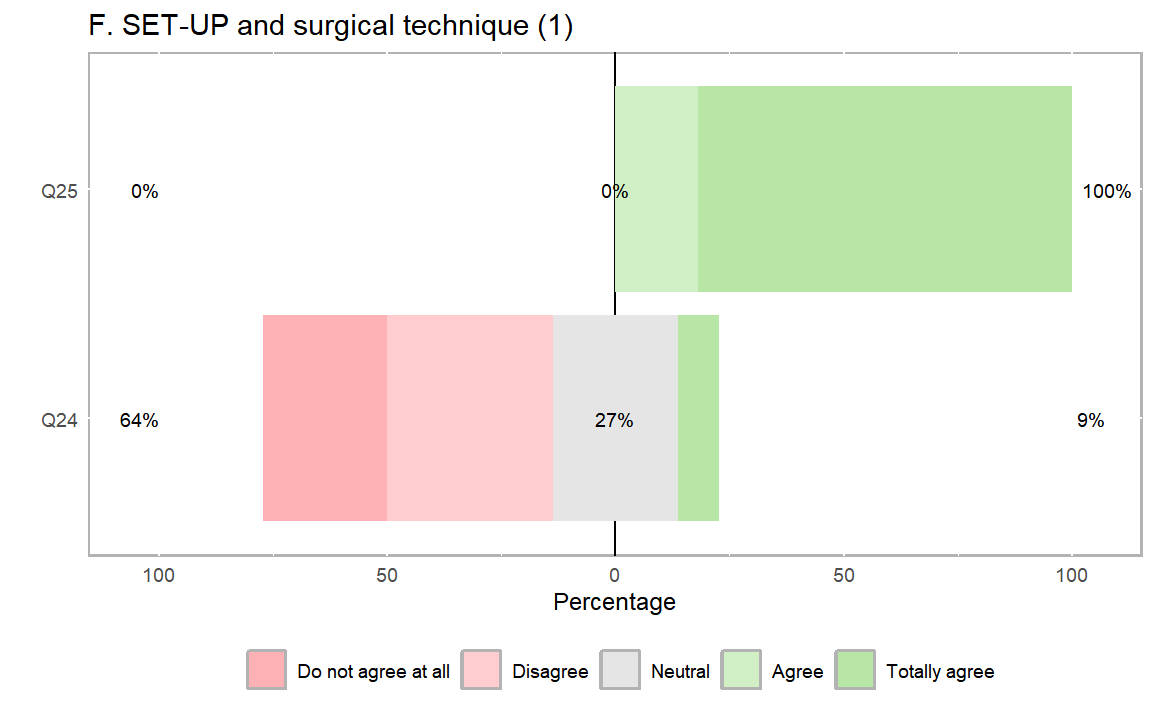


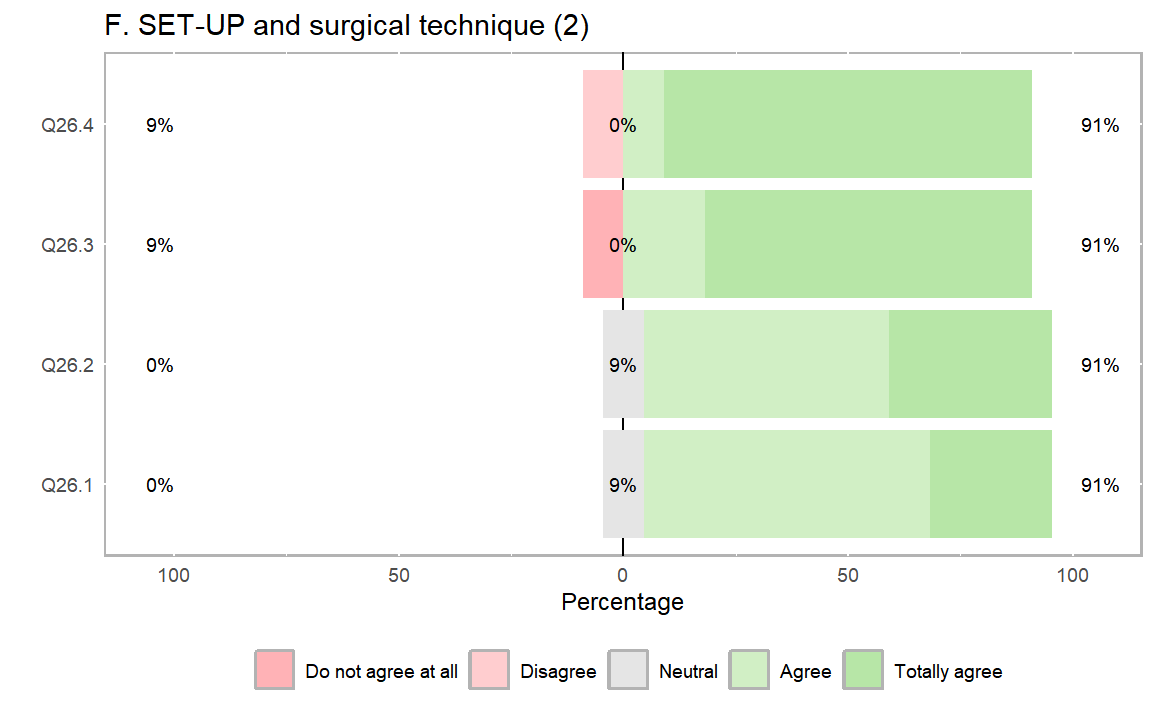


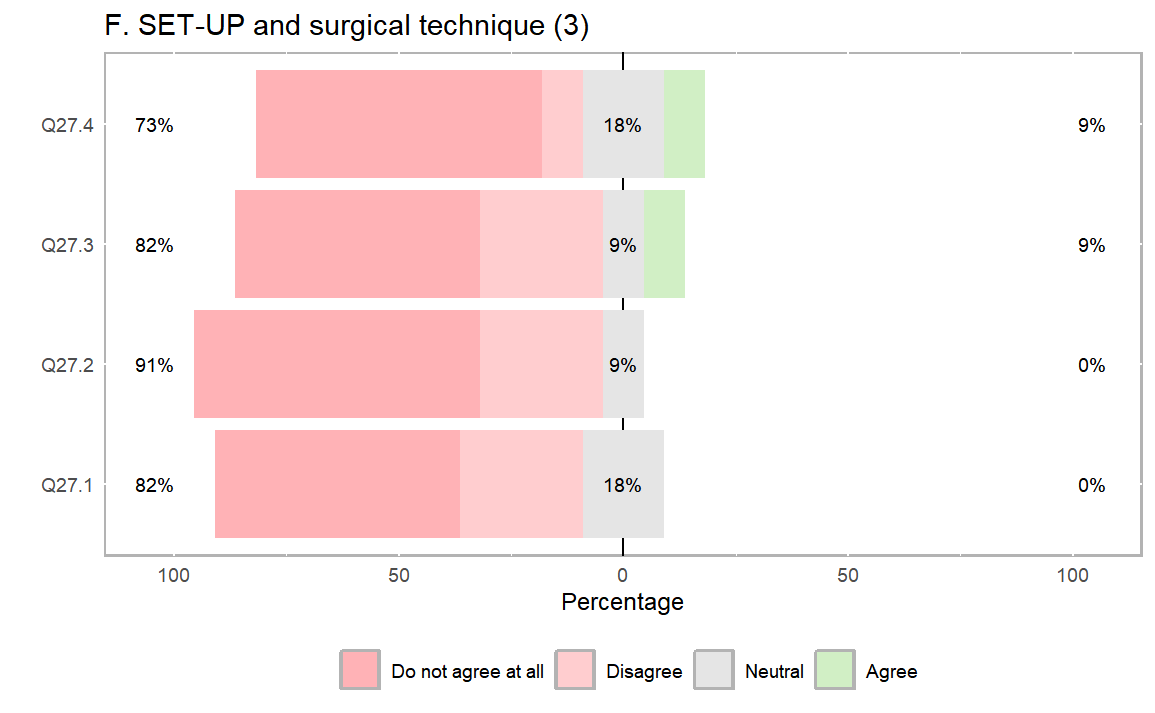


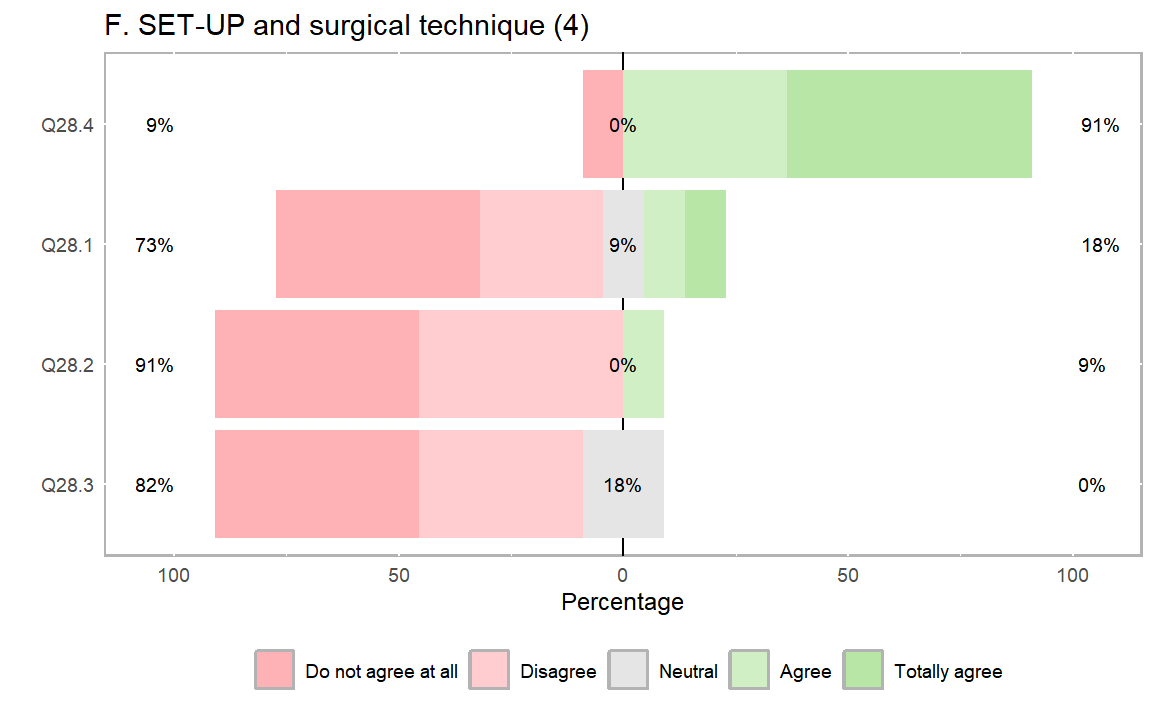


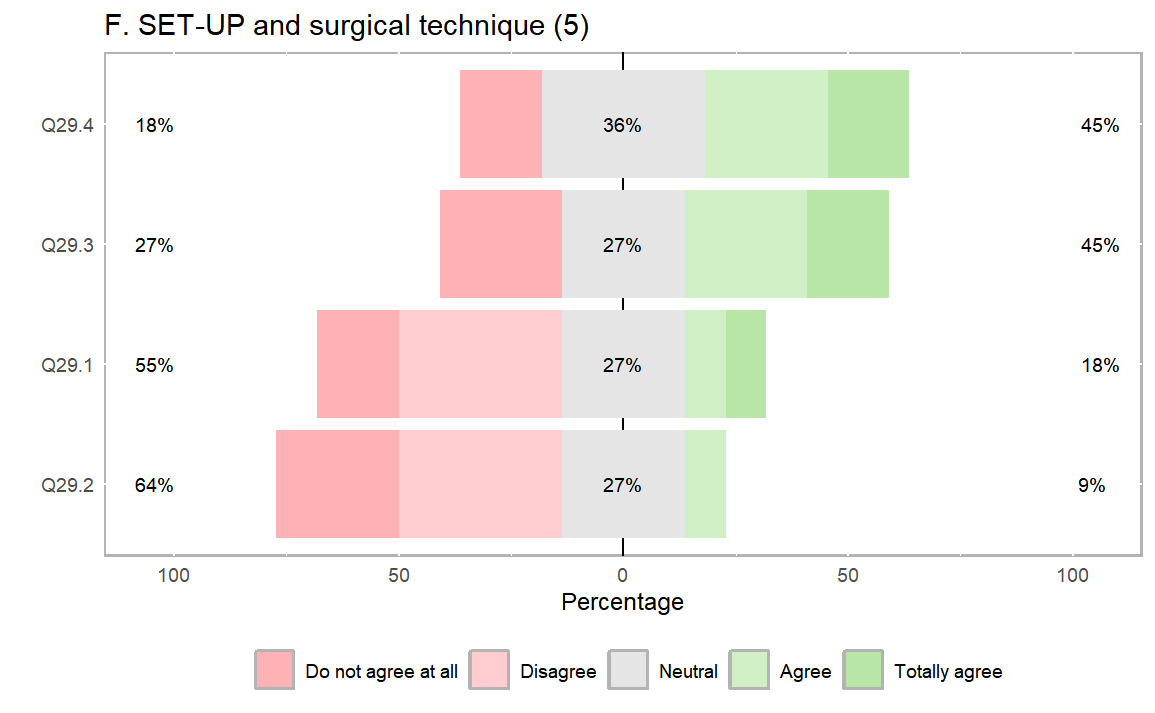


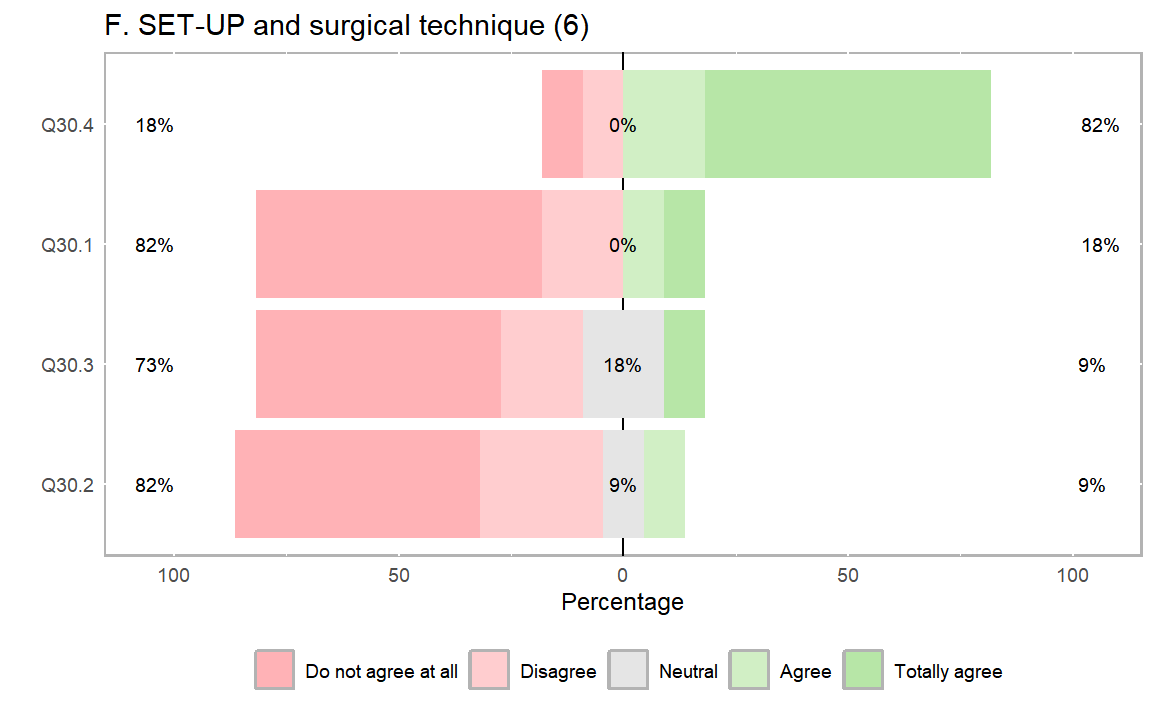

Supplement: Supplementary file 1 — (DOCX 225 KB) [file 384_2026_5118_MOESM1_ESM.docx]
